# L-tyrosine is a promising cancer vaccine adjuvant

**DOI:** 10.1186/2051-1426-3-S2-P440

**Published:** 2015-11-04

**Authors:** Hiep Khong, Meenu Sharma, Zhimin Dai, Manisha Singh, Yared Hailemichael, Willem Overwijk

**Affiliations:** 1 University of Texas - MD Anderson Cancer Center, Houston, TX, USA

## 

The choice of adjuvants is critical for any vaccine whether prophylactic or therapeutic. Our group previously showed that incomplete Freund's adjuvant, commonly used in different cancer vaccine trials, had many undesirable effects on T cell trafficking and function which directly translates into poor anti-tumor efficacy in preclinical murine model. Switching from oil-based (IFA) to water based (saline) vaccine eliminates those undesirable effects but at the cost of low specific T cell number. We therefore screened for adjuvants capable of inducing high T cell number with superior function and trafficking to the tumor. Here, we show that L-tyrosine particle is a very promising adjuvant for peptide based cancer vaccine. In combination with covax (including IL-2, anti-CD40 and imiquimod, a TLR-7 agonist), particle based L-tyrosine vaccine induces superior tumor specific CD8 T cell response, in terms of T cell number and function, resulting in remarkable anti-tumor efficacy. Mechanistic study shows that L-tyrosine particle extends antigen presentation time which is long enough for optimal T cell priming but not too long to induce a “grave yard” for the primed T cells. Understanding how the L-tyrosine particle works will provide more rationale for future vaccine adjuvant design.

